# The validity and clinical utility of axillary ultrasonography-guided fine needle aspiration biopsy in detection of nodal metastasis in early-stage breast cancer patients: a retrospective single-center experience

**DOI:** 10.55730/1300-0144.5419

**Published:** 2022-03-27

**Authors:** Filiz TAŞÇI, Yavuz METİN, Nurgül ORHAN METİN, Maksude Esra KADIOĞLU, Melih Gaffar GÖZÜKARA, Sibel KUL

**Affiliations:** 1 Department of Radiology, Faculty of Medicine, Recep Tayyip Erdoğan University, Rize, Turkey; 2 Department of Radiology, Faculty of Medicine, Ankara University, Ankara, Turkey; 3 Department of Radiology, Beytepe Murat Erdi Eker State Hospital, Ankara, Turkey; 4 Department of Radiology, Trabzon Kanuni Training and Research Hospital, Trabzon, Turkey; 5 Department of Public Health, Amasya Central Community Health Center, Amasya, Turkey; 6 Department of Radiology, Faculty of Medicine, Karadeniz Technical University, Trabzon, Turkey

**Keywords:** Axillary ultrasonography, axillary dissection, breast cancer, fine needle aspiration biopsy, sentinel lymph node biopsy

## Abstract

**Background/aim:**

Assessing the validity and clinical utility of axillary ultrasonography (AUS)-guided fine needle aspiration biopsy (FNAB) in detection of nodal metastasis during preoperative axillary investigation in comparison to the histopathologic diagnosis in early-stage breast cancer.

**Materials and methods:**

A total of 279 operated primary breast cancer patients (age: 55.3 ± 12.8, ranged 17–90 years) were included. Data on AUS findings at the time of initial diagnosis (first look AUS), second-look AUS findings performed by the breast radiologist during breast biopsy procedure and the AUS-guided FNAB findings were evaluated with respect to the final histopathology report obtained through axillary surgery via sentinel lymph node biopsy (SLNB) and/or axillary lymph node dissection (ALND). The diagnostic performance of each method in detecting metastatic ALNs were compared in terms of sensitivity, specificity, accuracy, positive predictive value (PPV), and negative predictive value (NPV).

**Results:**

The sensitivity, specificity, and accuracy of the first look AUS in detecting nodal metastasis were 64.56%, 86.78%, and 74.19% while the PPV and NPV were 86.44% and 65.22%, respectively. The sensitivity, specificity, and accuracy of the second-look AUS were 70.25%, 87.60%, and 77.78%, while PPV and NPV were 88.10% and 69.28%, respectively. The sensitivity, specificity, and accuracy of the second-look AUS guided FNAB were 89.19%, 73.33%, and 87.30%, while the PPV and NPV were 96.12% and 47.83%, respectively. The consideration of second-look AUS and finding of nodal metastasis in FNAB was associated with significantly higher likelihood of ALND (55.4% vs. 44.6%, p < 0.001) and lower likelihood of SLNB (34.7% vs. 65.3%, p < 0.001) compared to consideration of nonmetastatic ALN status. In 23 (22.3%) patients with positive findings on AUS-guided FNAB, SLNB was applied; 21 had positive results after surgical dissection, indicating that nearly 20% of patients had unnecessary SLNB.

**Conclusion:**

US-guided FNAB of suspicious ALNs is a simple, minimally invasive, and highly effective method for preoperative axillary staging in patients with invasive breast cancer avoiding the more invasive method SLNB and it enables the surgeon to proceed directly to ALND in positive cases.

## 1. Introduction

Axillary ultrasonography (AUS) is the primary imaging modality for evaluating axillary lymph nodes (ALNs) and its ability to identify the nodal metastases is considered critical for staging, prognosis, and treatment of patients with invasive breast cancer [1,2]. Preoperative axillary investigation via axillary ultrasonography (AUS)-guided fine needle aspiration biopsy (FNAB) has emerged as the most practical minimally invasive method in this regard, given its ability to confirm the presence of a metastasis in a suspicious ALN, and thus to avoid the unnecessary invasive sentinel lymph node biopsy (SLNB) and to allow node-positive patients to proceed directly to axillary lymph node dissection (ALND) as a single operative procedure [1–6].

In the clinical practice, while these “clinically node-positive” patients undergo ALND without undergoing SLNB, “clinically node-negative” patients undergo SLNB and those with negative results on SLNB has no further axillary dissection since there is no survival benefit for performing ALND in this setting [1,7]. SLNB was developed as a method for axillary staging in early invasive breast cancer which averts the unnecessary excision of ALNs in patients with clinically node-negative disease along with a high accuracy and a lower complication rate compared with ALND [8–11]. However, it is a complex laboratory technique with certain disadvantages such as anaphylactic reactions, frozen section difficulties, a longer operating time, and non-SLN skip metastasis along with no node involvement beyond SLNs in considerable portion breast cancer cases with positive SLNB have [10–14]. In addition, while patients with positive SLNB results traditionally undergo “completion” ALND, the role of ALND in these patients is also being reassessed [1,15].

Hence, the main goal of the radiologist is to determine the presence of metastatic disease in nonpalpable ALNs to reveal clinically node-positive patients before surgery by means of imaging with a positive predictive value that is high enough to be useful to the surgeon in selecting patients for upfront ALND or neo-adjuvant chemotherapy without undergoing SLNB [1,7,10,16,17].

In the context of alternative methods to replace SLNB with a decreasing role for axillary node dissection, US-guided FNAB has become a promising minimally invasive method in the accurate staging of the axilla with the preoperative detection of nodal metastasis, while a need for larger-scale and longer–follow-up comparative studies has been emphasized [13,18,19].

This study aimed to assess the validity and clinical utility of AUS- and US-guided FNAB in detection of nodal metastasis during preoperative axillary investigation in comparison to the final histopathologic results in early-stage breast cancer patients and to determine its impact on the selection of ALND and/or SLNB by the surgeon.

## 2. Materials and methods

### 2.1. Study population

A total of 279 operated primary breast cancer patients (mean ± SD age: 55.3 ± 12.8 years, ranged 17–90 years) were included in this retrospective study conducted between September 2014 and December 2018. Patients who received preoperative neoadjuvant chemotherapy and those without axillary histopathology report were excluded from the study.

The study was conducted in accordance with the ethical principles stated in the “Declaration of Helsinki” and approved by the institutional ethics committee along with the permission for the use of patient data for publication purposes.

### 2.2. Assessments

Second-look AUS was performed during histopathological examination of the primary breast tumor and FNAB was performed to all patients with suspected lymph nodes. In our study; lymph nodes with a short diameter greater than 1 cm, a round shape, an indistinct fatty hilum, and more than 3 mm of concentric or focal cortical thickening were considered suspicious. Afterwards, all patients were gone through axillary surgery for the final histopathologic report. The patients with malignant FNAB underwent SLNB and/or ALND at the time of definitive surgery, while those with benign, suspicious, or insufficient FNABs underwent SLNB using blue-dye and/or radio-colloid injection.

Data on AUS findings at the time of initial diagnosis, second-look AUS findings performed by the breast radiologist to consider the need for axillary biopsy during the primary breast tumor biopsy procedure, and the FNAB findings were evaluated with respect to the final operative histopathologic results. The diagnostic performance of each method in detecting metastatic axillary lymph nodes were compared in terms of sensitivity, specificity, accuracy, positive predictive value (PPV), and negative predictive value (NPV). By comparing the FNAB with final histopathologic result, the rate of unnecessary SLNB was estimated.

### 2.3. Statistical analysis

Statistical analysis was made using IBM SPSS Statistics for Windows, version 23.0 (IBM Corp., Armonk, NY). Pearson’s chi-squared test and Fisher’s exact test were used for the comparison of categorical data. Data were expressed as percent (%) and 95% confidence interval (CI) where appropriate. p < 0.05 was considered statistically significant.

## 3. Results

### 3.1. Baseline characteristics and diagnostic work-up

Invasive ductal carcinoma was the most common tumor subtype, while the rates of luminal A and luminal B molecular subtypes were 41.6% each. Nonbreast-conserving surgery was applied in 67.0% of patients, while axillary lymph node dissection and sentinel lymph node dissection were applied in 48.4% and 27.6% of patients, respectively (Table 1).

The nodal metastasis was considered in 42.3% of patients by the first-look axillary US, in 45.1% of patients by second-look AUS. Metastatic LAP was detected in 36.9% of patients who underwent FNAB. After axillary SLNB and/or ALND, the presence of metastatic LAP was histopathologically proven in 56.6% of patients (Table 1).

Final histopathologic report revealed the number of benign and metastatic lymph node yield to be mean 8.84 ± 6.15 (ranged 1 to 31) and 3.68 ± 2.69 (ranged 1 to 13), respectively. Mean size of metastatic lymph nodes was 16.76 ± 7.81 (ranged 3–45) mm.

### 3.2. The diagnostic performance of first-look AUS with respect to second-look AUS-guided FNAB decision

In 103 of 118 ALNs suspected of metastatic involvement in the first-look AUS, the second-look AUS was required with decision of FNAB. In 23 of 161 patients with ALNs considered to be normal in the first-look AUS, second-look AUS was required with decision of FNAB (Table 2).

The sensitivity, specificity and accuracy of the first-look AUS in detecting the nodal metastasis were 81.75%, 90.20%, and 86.38%, while the PPV and NPV were 87.29% and 85.71%, respectively (Table 2).

### 3.3. The diagnostic performance of second-look AUS with respect to FNAB findings

Of 126 patients who underwent FNAB after second-look AUS, FNAB findings revealed nodal metastasis in 56.5% of cases considered to be nonmetastatic in the second-look AUS, while in 87.4% of cases considered to be metastatic in the second-look AUS (Table 2).

The sensitivity, specificity and accuracy of the second-look AUS in detecting nodal metastasis were 87.38%, 43.48%, and 79.37%, while the PPV and NPV were 87.38% and 43.48%, respectively (Table 2).

### 3.4. Diagnostic performance of first-look AUS, second-look AUS and FNAB with respect to definite operative histology

The definite operative histology confirmed nodal metastasis in 86.4% of 118 patients with suspicious ALNs on initial AUS, in 88.1% of 126 patients with suspicious ALNs in second-look AUS, and in 96.1% of 103 patients with positive findings on FNAB (Table 3).

The definite operative histology confirmed nodal metastasis in 34.8% of 161 cases considered to be not metastatic in the first-look AUS, in 30.7% of 153 cases considered not necessary to be evaluated via FNAB in second-look AUS, and in 52.2% of 23 cases considered to be not metastatic in FNAB (Table 3).

When compared to negative findings, the likelihood of positive findings suggestive of metastasis in the first-look AUS, second-look AUS, and FNAB was significantly higher to reveal a nodal metastasis on definite operative histology, overall (p < 0.001 for each) as well as in luminal A (p < 0.001), luminal B (p < 0.001), triple negative (p = 0.009) subcategories (Table 3).

When compared to definite operative histology; the sensitivity, specificity, and accuracy of the first-look AUS in detecting nodal metastasis were 64.56%, 86.78%, and 74.19%, while the PPV and NPV were 86.44% and 65.22%, respectively. The sensitivity, specificity, and accuracy of the second-look AUS were 70.25%, 87.60%, and 77.78%, while the PPV and NPV were 88.10% and 69.28%, respectively. The sensitivity, specificity and accuracy of the second-look AUS guided FNAB were 89.19%, 73.33%, and 87.30%, while the PPV and NPV were 96.12% and 47.83%, respectively (Table 3).

### 3.5. The value of first-look AUS in selection of surgery type and LN dissection method

Nonbreast-conserving surgery was significantly more commonly selected for cases considered vs. not considered to be metastatic during first-look AUS (74.6% vs. 61.5%, p = 0.022). The sensitivity, specificity, and accuracy of first-look AUS in selecting the surgery type were 44.06%, 67.39%, and 53.76%, respectively (Table 4).

The consideration of nodal metastasis during first-look AUS was associated with significantly higher likelihood of ALND (70.3% vs. 32.3%, p < 0.001) and lower likelihood of SLNB (5.1% vs. 44.1%, p < 0.001) compared to consideration of nonmetastatic ALN status (Table 4).

### 3.6. The value of FNAB in selection of LN dissection method

The consideration of second-look AUS and finding of nodal metastasis in FNAB was associated with significantly higher likelihood of ALND (55.4% vs. 44.6%, p < 0.001) and lower likelihood of SLNB (34.7% vs. 65.3%, p < 0.001) compared to consideration of nonmetastatic ALN status (Table 4).

### 3.7. Unnecessary SLNB rate

In 23 (22.3%) of 103 patients with positive findings on US guided FNAB, SLNB was applied. Of 23 patients with SLNB, 21 (91.03%) had positive results after surgical dissection, indicating that nearly 20% of patients had unnecessary SLNB.

## 4. Discussion

The nodal metastasis rates obtained via first-look AUS (42.3%), second-look AUS-guided FNAB (36.9%) and the definite operative histology (56.6%) in our study were consistent with the definite histopathology report in 86.4%, 88.1%, and 96.1% of initial predictions, respectively; while US-guided FNAB was positive in 70.3% of cases with definite pathology.

Similarly, in the study of Leenders et al., it was reported that AUS showed suspicious lymph nodes in 28.4% cases, FNAB showed axillary metastases in 32.7% of these LNs, and the final histological analysis confirmed metastatic disease in 37.3% of these suspected LNs [20]. Other studies also reported that the US-guided FNAB identified the structurally abnormal ALNs in 42.0% to 63.9% of patients with positive final pathology [11,21–24]. Hence, our findings support that US-guided lymph node sampling via FNAB is a quick, well tolerated and indispensable method of confirming the presence of a metastasis in a node suspicious on imaging before the patient undergoes ALND [1,6,16].

The sensitivity and specificity of second-look AUS (70.25% and 87.60%) and FNAB (89.19% and 73.33%) in the current study are in line with the reported ranges of moderate sensitivity (25% to 87%) and a higher specificity (77%–100%) for AUS-FNAB in the literature, supporting that both sensitivity and specificity of AUS increases when combined with FNAB and with selection of only the ALNs deemed to be suspicious on AUS for aspiration [4,16,17,19,20,25,26].

However, considering the definite operative histology as a reference, the second-look AUS and FNAB revealed false-negative results in 30.7% and 52.2% of our cases, respectively. Hence, AUS-guided FNAB was associated a PPV of 96.12% but an NPV of 47.83%, supporting the previously reported low NPV (ranged 59.3% to 73.0%) and high false negativity (ranged 19.4% to 31.8%) of this method in detecting ALN metastases [10,11,13,17,20,27,28]. Given that the negative AUS-FNAB revealed nonmetastatic axillae only in half of our patients, our findings seem to support that AUS-FNAB alone is not likely to be relied upon in axillary investigation to replace SLNB for the time being due to moderate sensitivity and the high false-negative rate [17,20].

The sampling error, micrometastasis, and errors in radiologic and pathologic assessment are considered to be the possible causes of false-negative results [11,17,18,22,29,30], while the sensitivity of AUS-FNAB is also suggested to increase with tumor size [5,10,31–33]. The average size of metastatic lymph nodes (16.76 mm, ranged 3 to 45 mm) in the current study seems notable in this regard, given the higher probability of metastases >0.5 mm than small metastases (<0.5 mm) to be detected by FNAB (%93 vs. 44%) [30]. Indeed, while the false-negativity of AUS-FNAB is especially detected in lymph nodes with micrometastases or isolated tumor cells, the necessity of ALND in such situations remains also controversial [17].

Notably, in a past study with 27 early-stage breast cancer patients, FNAB in comparison with SLNB was reported to have a sensitivity, specificity, PPV and NPV of 45%, 100%, 100%, and 73%, respectively [13]. The authors concluded the similar specificity of FNAB-based and SLNB-based ALN cytology in the presence of ALN metastases, whereas lower sensitivity of FNAB than SLNB when lymph node cytology is negative, indicating that negative AUS-FNAB results do not rule out the metastatic implants [13,34].

In contrary, for patients with preoperative identification of positive AUS and AUS-guided FNAB, the need for SLNB is considered likely to be eliminated; thus, AUS-guided positive FNAB is suggested to allow patients to be triaged to ALND, bypassing the potentially unnecessary SLNB [1,11,35].

Likewise, our findings indicated the increased likelihood of ALND rather than SNLB to be considered for a ALN found metastatic on a second-look USG guided FNAB, while in nearly 20% of patients who had been initially positive for AUS-FNAB, the SLNB revealed positive findings. Hence, indicating the primary role of radiologist by providing accurate AUS guidance for FNAB, our findings emphasize that the unnecessary SNLBs can be avoided in at least one fifth of node-positive patients with primary breast cancer via preoperative AUS-FNAB-based axillary investigation. This supports the consideration of node positive group to benefit most from axillary FNAB with consequent reduction of SLNB [6,17,20,36] and that positive AUS-FNAB can spare SLND and enable the surgeon to proceed directly with ALND in 8%–28% of breast cancer patients [19,20,25].

In a past study, the authors reported US-guided FNAB positivity in 821 of 1152 patients which resulted in avoiding 11.7% of patients to undergo needless SLNB [37]. Likewise, a metaanalysis of 31 studies on 2397 AUS-guided biopsies (FNAC and core biopsies) of ALNs in breast cancer patients revealed a sensitivity of 75.0% and a specificity of 98.5% along with 19.8% rate of women triaged directly to ALND [38]. Also, in a systematic review of studies in breast cancer patients, the unnecessary SLNBs were reported to be avoided in 1%–28% of patients with preoperative axillary FNAB [39].

In a prospective study including 100 female patients with breast cancer, the overall AUS-FNAB sensitivity was reported to be 79.4% with PPV and NPV of 100% and 69.5%, respectively, while the AUS-FNAB sensitivity was 0% for lymph nodes with normal sonographic features, 80% for indeterminate lymph nodes and 90.5% for suspicious lymph nodes [2]. The authors concluded that AUS should be included in the preoperative staging of all patients with invasive breast cancer, while addition of FNAB to AUS in cases of lymph nodes suspicious for malignancy could avoid SLNB in 54% of cases, significantly shortening the time interval to definitive therapy [2].

The increased likelihood of ALND rather than SNLB in cases with second-look AUS-guided FNAB positivity compared to those with negative FNAB findings in the current study seems notable given the consideration of an abnormal AUS and positive FNAB to correlate with patients eligible for ALND as well as with a higher nodal burden on final pathology compared with patients with metastasis identified on SLNB [19]. Indeed, while AUS-guided FNAB has been considered to be useful in diagnosing lymph node metastases and triaging breast cancer patients directly to ALND, there is a trend toward less aggressive axillary surgery with ongoing controversy regarding the role of ALND in sentinel node-positive women due to emerging evidence indicating no survival benefit of completion ALND in early breast cancer patients with node-positive disease [1,20,40–44].

Given its association with lower false-negative rate than the AUS-FNAB, the AUS-guided core needle biopsy (CNB) has also become an increasingly more popular approach of axillary staging in breast cancer patients which avoids SLNB and a second trip to the operating room [1]. Nonetheless, in a metaanalysis of 67 studies on US-FNAB and CNB of ALNs in patients with breast cancer, a diagnostic test accuracy revealed that CNB showed higher sensitivity than US-FNAC (0.849 vs. 0.760), while there was no difference in specificity between US-FNAC and CNB (0.997 vs. 1.000) [45]. The authors concluded that both US-FNAC and CNB are useful in preoperative assessments of ALNs in patients with breast cancer [45].

The FNAB positivity in 56.5% of AUS negative cases in the current study seems also notable given that AUS-FNAB is performed for only patients with suspicious ALN on AUS and the use of US-FNAB for nonsuspicious ALNs is considered to be associated with classification of these cases as true negatives [45]. Hence, studies addressing the comparison of the diagnostic accuracy between suspicious and nonsuspicious subgroups in AUS are considered to be useful for improving the diagnostic accuracy of preoperative assessments of ALNs and lowering the false-negative rate of AUS and AUS-FNAB [45].

Notably, in a study among 101 patients including those with positive AUS-FNAB and corresponding ALND (n = 65) and those with negative US-FNA with corresponding ALND/SLNB (n = 36), 43% of patients in the positive US-FNA group were reported to have two or fewer positive lymph nodes upon ALND pathologic examination (indicating the risk of overtreatment) [46]. The authors also reported the NPV of detecting axillary disease was 83.3% in the AUS-FNAB negative group, indicating the possibility of undertreatment in 16.7% of patients [46]. The comparative analysis between US-FNA and SLNB showed that performing US-FNA resulted in a reduction in SLNB reaching 40%, avoiding an additional surgical procedure, and reducing the cost by up to 20% [4,46–48].

The retrospective single-center design of the present study seems to be the major limitation that prevents the establishing temporality between the cause and effect as well as generalizing our findings to overall breast cancer population.

In conclusion, US-guided FNAB of suspicious ALNs is a simple, minimally invasive and highly effective method for preoperative axillary staging in patients with invasive breast cancer that can identify those patients with high metastatic nodal burden and thus potentially avoid SLNB and enable the surgeon to proceed directly to ALND in positive cases.

## Figures and Tables

**Table 1:** Baseline characteristics and diagnostic work-up (n = 279).

Tumor subtype, n(%)	
Invasive ductal carcinoma	224(80.3)
Mucinous carcinoma	7(2.5)
Invasive lobular carcinoma	8(2.9)
Invasive breast cancer	34(12.2)
Tubular cancer	3(1.1)
Ductal carcinoma in situ	2(0.7)
Solid papillary carcinoma	1(0.4)
**Molecular sub-type, n(%)**	
Luminal A	116(41.6)
Luminal B	116(41.6)
Triple negative	21(7.5)
Her-2 +	26(9.3)
**Surgery type, n(%)**	
Breast conserving surgery	92(33.0)
Nonbreast conserving surgery	187(67.0)
**Lymph node dissection type, n(%)**	
Sentinel lymph node dissection	77(27.6)
Axillary lymph node dissection	135(48.4)
Both	67(24.0)
**First-look axillary US, n(%)**	
Metastatic lymph node (−)	161(57.7)
Metastatic lymph node (+)	118(42.3)
**Second look axillary US, n(%)**	
No need for biopsy	153(54.8)
FNAB performed	126(45.1)
Metastasis (−)	23(8.2)
Metastasis (+)	103(36.9)
**Definite operative histology**	
Metastasis (−)	121(43.4)
Metastasis (+)	158(56.6)

**Table 2:** Diagnostic performance of first-look axillary US with respect to second-look axillary US-guided FNAB decision and performance of second-look US with respect to FNAB findings.

	Second-look axillary USG, n(%)			Diagnostic performance (%, 95% CI [min–max])				
	No biopsy	FNAB is taken	p	PPV	NPV	Sensitivity	Specificity	Accuracy
**First-look axillary AUS (n = 279)**								
Metastatic LN (−) (n = 161)	138 (85.7)	23 (14.3)	**<0.001** 1	87.29 (80.83–91.79)	85.71 (80.51–89.71)	81.75 (73.88–88.06)	90.20 (84.35–94.41)	86.38 (81.79–90.18)
Metastatic LN (+) (n = 118)	15 (12.7)	103 (87.3)						
	**FNAB findings, n(%)**			**Diagnostic performance (%, 95% CI [min-max])**				
	Metastatic LN (−)	Metastatic LN (+)	**p**	PPV	NPV	Sensitivity	Specificity	Accuracy
**Second-look AUS (n = 126)**								
Metastatic LN (−) (n = 23)	10 (43.5)	13 (56.5)	**0.002** 2	87.38 (82.76–90.89)	43.48 (27.85–60.52)	87.38 (79.38–93.11)	43.48 (23.19–65.51)	79.37 (71.25–86.06)
Metastatic LN (+) (n = 123)	13 (12.6)	90 (87.4)						

Note: AUS: axillary ultrasonography; LN: lymph node; FNAB: fine needle aspiration biopsy; PPV: positive predictive value; NPV: negative predictive value;

1 Pearson’s chi-squared test;

2 Fisher’s exact test.

**Table 3:** Diagnostic performance of first-look AUS, second-look AUS and FNAB with respect to definite operative histology.

	Definite operative histology			Diagnostic performance (%, 95% CI [min–max])				
	Metastatic LN (−)	Metastatic LN (+)	p	PPV	NPV	Sensitivity	Specificity	Accuracy
**First-look AUS (n = 279)**								
Metastatic LN −	105 (65.2)	56 (34.8)	**<0.001** 1	86.44 (79.92–91.08)	65.22 (60.04–70.06)	64.56 (56.56–71.99)	86.78 (79.42–92.25)	74.19 (68.64–79.23)
Metastatic LN +	16 (13.6)	102 (86.4)						
**Second-look AUS (n = 279)**								
No biopsy	106 (69.3)	47 (30.7)	**<0.001** 1	88.10 (80.01–92.31)	69.28 (63.75–74.31)	70.25 (62.47–77.25)	87.60 (80.38–92.89)	77.78 (72.44–82.52)
Biopsy is taken (metastasis)	15 (11.9)	111 (88.1)						
**FNAB (n = 126)**								
Metastatic LN −	11 (47.8)	12 (52.2)	**<0.001** 1	96.12 (91.43–98.29)	47.83 (33.13–62.91)	89.19 (81.19–94.29)	73.33 (44.90–92.21)	87.30 (80.20–92.56
Metastatic LN +	4 (3.9)	99 (96.1)						
**Luminal A- ALN prediction (n = 116)**								
Metastatic LN −	53 (75.7)	17 (24.3)	**<0.001** 1	84.78 (73.11–91.95)	75.71 (67.48–82.41)	69.64 (55.90–81.22)	88.33 (77.43–95.18)	79.31 (70.80–86.27)
Metastatic LN +	7 (15.2)	39 (84.8)						
**Luminal B- ALN prediction (n = 116)**								
Metastatic LN −	39 (57.4)	29 (42.6)	**<0.001** 1	93.75 (82.23–97.84)	57.35 (50.01–64.39)	60.81 (48.77–71.96)	92.86 (80.52–98.50)	72.41 (63.34–80.30)
Metastatic LN +	3 (6.3)	45 (93.8)						
**Triple negative**- **A LN prediction (n = 21)**								
Metastatic LN −	7 (77.8)	2 (22.2)	**0.009** 2	83.33 (58.93–94.57)	77.78 (48.51–92.86)	83.33 (51.59–97.91)	77.78 (39.99–97.19)	80.95 (58.09–94.55)
Metastatic LN +	2 (16.7)	10 (83.3)						
**Her-2 positive (n = 26)**								
Metastatic LN −	6 (42.9)	8 (57.1)	0.701^2^	–	–	–	–	–
Metastatic LN +	4 (33.3)	8 (66.7)						

Note: AUS: axillary ultrasonography; ALN: axillary lymph node; FNAB: fine needle aspiration biopsy; PPV: positive predictive value; NPV: negative predictive value;

1 Pearson’s chi-squared test;

2 Fisher’s exact test.

**Table 4:** The value of first-look AUS and second-look AUS-guided FNAB in selection of surgery and/or LN dissection method.

	Type of surgery, n (%)							
	Breast conserving	Non-breast conserving	p	Diagnostic performance (%, 95% CI [min–max])				
				PPV	NPV	Sensitivity	Specificity	Accuracy
**First-look AUS (n = 279)**								
Metastatic LN (−), no biopsy (n = 161)	62 (38.5)	99 (61.5)	**<0.022** 1	74.58 (67.82–80.33)	38.51 (33.98–43.25)	44.06 (39.74–54.48)	67.39 (56.82–76.80)	53.76 (47.72–59.73)
Metastatic LN (+) (n = 118)	30 (25.4)	88 (74.6)^a^						
	**LN dissection method, n(%)**							
	SLNB	ALND	**p**					
**First-look AUS (n≠** **2** **279)**								
Metastatic LN (−) (n≠^2^161)	109 (54.8)	90 (45.2)	**<0.001** 1	76.19 (70.05–81.41)	54.77 (50.3–59.17)	55.45 (48.31–62.42)	75.69 (67.85–82.45)	63.87 (58.56–68.94)
Metastatic LN (+) (n≠^2^118)	35 (23.8)	112 (76.2)^a^						
**Second look-US and FNAB findings**								
Metastatic LN (−), no biopsy (n≠^2^176)	121 (54.5)	101 (45.5)	**<0.001** 1	81.45 (74.66–86.75)	54.50 (50.64–58.32)	50.00 (42.90–57.10)	84.03 (77.00–89.60)	64.16 (58.86–69.22)
Metastatic LN (+) (n≠^2^103)	23 (18.5 )	101 (81.5)^a^						

Note: AUS: axillary ultrasonography; SLNB: sentinel lymph node biopsy; ALND: axillary lymph node dissection; FNAB: fine needle aspiration biopsy; PPV: positive predictive value; NPV: negative predictive value;

a Used as true positive;

1 Pearson’s chi-squared test;

2 Some patients have both surgeries.
